# Efficient manganese luminescence induced by Ce^3+^-Mn^2+ ^energy transfer in rare earth fluoride and phosphate nanocrystals

**DOI:** 10.1186/1556-276X-6-119

**Published:** 2011-02-04

**Authors:** Yun Ding, Liang-Bo Liang, Min Li, Ding-Fei He, Liang Xu, Pan Wang, Xue-Feng Yu

**Affiliations:** 1 Department of Physics, Key Laboratory of Artificial Micro- and Nano-structures of Ministry of Education and School of Physics and Technology, Wuhan University, Luoshi Road, Wuhan 430072, China

## Abstract

Manganese materials with attractive optical properties have been proposed for applications in such areas as photonics, light-emitting diodes, and bioimaging. In this paper, we have demonstrated multicolor Mn^2+ ^luminescence in the visible region by controlling Ce^3+^-Mn^2+ ^energy transfer in rare earth nanocrystals [NCs]. CeF_3 _and CePO_4 _NCs doped with Mn^2+ ^have been prepared and can be well dispersed in aqueous solutions. Under ultraviolet light excitation, both the CeF_3_:Mn and CePO_4_:Mn NCs exhibit Mn^2+ ^luminescence, yet their output colors are green and orange, respectively. By optimizing Mn^2+ ^doping concentrations, Mn^2+ ^luminescence quantum efficiency and Ce^3+^-Mn^2+ ^energy transfer efficiency can respectively reach 14% and 60% in the CeF_3_:Mn NCs.

## Introduction

The preparation of fluorescent nanomaterials continues to be actively pursued in the past decades. The potentially broad applicability and high technological promise of the fluorescent nanomaterials arise from their intrinsically intriguing optical properties, which are expected to pale their bulk counterparts [1-4]. Particularly, controllable energy transfer in the nanomaterials has been receiving great interest because it leads luminescence signals to outstanding selectivity and high sensitivity, which are important factors for optoelectronics and optical sensors [5].

Great efforts have been devoted to Mn^2+^-doped semiconductor nanocrystals [NCs] due to their efficient sensitized luminescence [6,7]. When incorporating Mn^2+ ^ions in a quantum-confined semiconductor particle, the Mn^2+ ^ions can act as recombination centers for the excited electron-hole pairs and result in characteristic Mn^2+ ^(^4^T_1_-^6^A_1_)-based fluorescence. Compared with the undoped materials, the Mn^2+^-doped semiconductor NCs often have higher fluorescence efficiency, better photochemical stability, and prolonged fluorescence lifetime. Therefore, such Mn^2+^-doped NCs have recently been proposed as bioimaging agents [8,9] and recombination centers in electroluminescent devices [10,11]. They may even find applications in future spin-based information processing devices [12,13] and have been examined as models for magnetic polarons [14]. Moreover, as emission centers, Mn^2+ ^ions can be used for the synthesis of long persistent phosphors [15,16], and white-light ultraviolet light-emitting diodes [17], when doped in inorganic host materials (such as silicate, aluminate, and fluoride).

Rare earth ions (such as Ce^3+ ^and Eu^2+^) have been commonly used as sensitizers to improve Mn^2+ ^fluorescence efficiency in bulk materials [18-20]. Typically, the efficient room temperature [RT] luminescence were reported in the Mn^2+^, Ce^3+ ^co-doped CaF_2 _single crystal and other matrixes, which were assigned to the energy transfer from the Ce^3+ ^sensitizers to the Mn^2+ ^acceptors through an electric quadrupole short-range interaction in the formed Ce^3+^-Mn^2+ ^clusters [18]. However, a portion of isolated Ce^3+ ^and Mn^2+ ^ions which are randomly dispersed in the host usually causes a low Ce^3+^-Mn^2+ ^energy transfer efficiency.

In this work, we have synthesized the CeF_3_:Mn and CePO_4_:Mn NCs and investigated the Ce-Mn energy transfer in these representative rare earth NCs. Upon UV light excitation, both the CeF_3_:Mn and CePO_4_:Mn show bright Mn^2+ ^luminescence in the visible region. Their fluorescence output colors, however, are quite different owing to different host crystal structures. The optimum Mn^2+ ^doping concentration has been found at which the Mn^2+ ^luminescence quantum efficiency and Ce^3+^-Mn^2+ ^energy transfer efficiency peak at 14% and 60% in the CeF_3_:Mn NCs, respectively.

## Experimental section

### Materials

Reagents MnCl_2 _(>99%), TbCl_3 _(>99%), CeCl_3 _(>99%), NH_4_F (>99%), and H_3_PO_4 _(>85%) were obtained from Sinopharm Chemical Reagent Co., Ltd. (Beijing, China). Polyethylenimine [PEI] (branched polymer (-NHCH_2_CH_2_-)_*x *_(-N(CH_2_CH_2_NH_2_)CH_2_CH_2_-)_*y*_) was purchased from Sigma-Aldrich (St. Louis, MO, USA). All reagents were used as received without further purification.

### Synthesis of CeF_3_:Mn nanocrystals

CeF_3 _NCs were synthesized using a modified method reported previously [21]. In a typical procedure, *x *mL of 0.2 M MnCl_2 _and (0.2 - *x*) mL of 0.2 M CeCl_3 _were added to 15 mL of ethanol with 5 mL of PEI solution (5 wt.%). After stirring for 30 min, an appropriate amount of NH_4_F was charged. The well-agitated solution was then transferred to a Teflon-lined autoclave and subsequently heated at 200°C for 2 h. After cooling down, the product was isolated by centrifugation, washed with ethanol and deionized water several times, and dried in vacuum.

### Synthesis of CePO_4_:Mn nanocrystals

In a typical procedure, *x *mL of 0.2 M MnCl_2 _and (12 - *x*) mL of 0.2 M CeCl_3 _were mixed. The mixture was agitated for 10 min, then charged with 5 mL of 0.5 M H_3_PO4, and eventually placed under ultrasonic irradiation for 2 h. All ultrasonic irradiations were performed in a water bath with an ultrasonic generator (100 W, 40 kHz; Kunshan Ultrasonic Instrument Co., Shanghai, China). The particles were obtained by centrifugation, washed with ethanol and deionized water several times, and dried in vacuum.

### Physical and optical measurements

The transmission electron microscopy [TEM] measurements were carried out on a JEOL 2010 HT transmission electron microscope (operated at 200 kV). X-ray diffraction [XRD] analyses were performed on a Bruker D8-advance X-ray diffractometer with Cu Kα irradiation (*λ *= 1.5406 Å). The absorption spectra were obtained with a Varian Cary 5000 UV/Vis/NIR spectrophotometer. The photoluminescence [PL] and PL excitation [PLE] spectra were recorded by a Hitachi F-4500 fluorescence spectrophotometer with a Xe lamp as the excitation source.

## Results and discussion

### Morphology and structure

**Figure 1:**
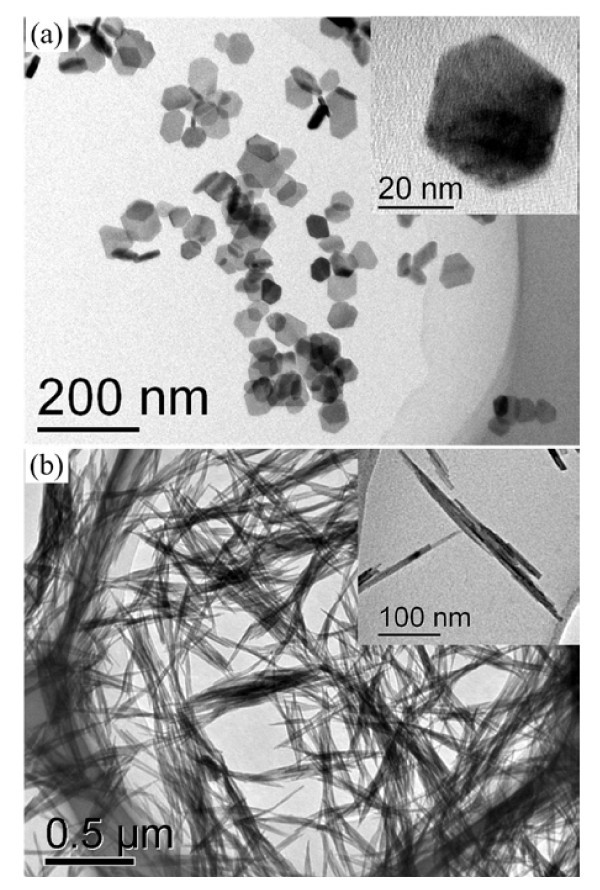
**TEM images**. TEM images of CeF_3_:Mn (**a**) and CePO_4_:Mn (**b**) NCs.

Both the CeF_3_:Mn and the CePO_4_:Mn NCs were synthesized by effective hydrothermal processes. The prepared CeF_3_:Mn NCs are shaped as hexagonal plates with average sizes of ~25 nm, as shown by the TEM image in Figure 1a. Figure 1b demonstrates CePO_4_:Mn nanowires with an average diameter of ~8 nm and an average length of ~400 nm.

**Figure 2:**
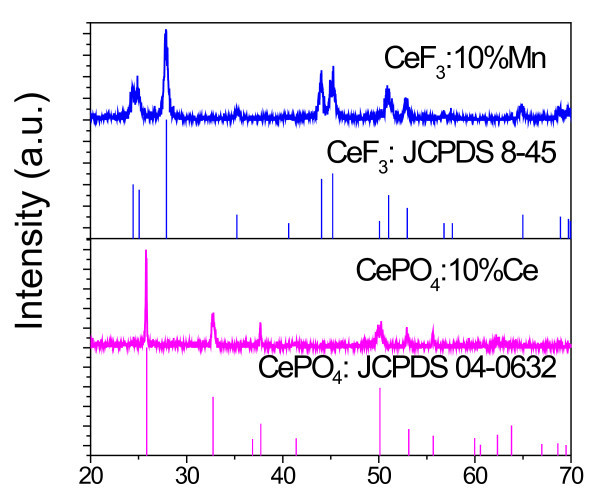
**XRD spectra**. XRD spectra of CeF_3_:Mn and CePO_4_:Mn NCs.

Figure 2 shows XRD spectra of CeF_3_:Mn and CePO_4_:Mn NCs. The XRD pattern of the CeF_3_:Mn NCs shows that all the peak positions are in good agreement with the literature data of the hexagonal CeF_3 _crystal, and the peak positions exhibited by the CePO_4_:Mn NCs are well indexed in accord with the hexagonal CePO_4 _crystal, revealing high crystallinity of these two kinds of products.

### Absorption spectra

**Figure 3:**
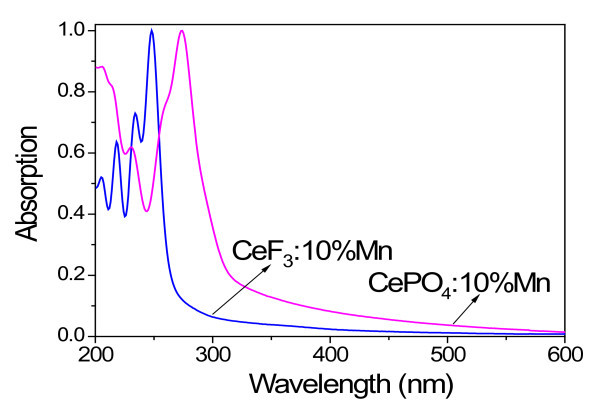
**Absorption spectra attributed to electronic transitions**. Absorption spectra of CeF_3_:Mn and CePO_4_:Mn NCs.

As shown in Figure 3, the CeF_3_:Mn NCs exhibit four absorption peaks located at 248, 235, 218, and 205 nm, which are attributed to the electronic transitions from the ground state to different 5*d *states of the Ce^3+ ^ions. The above absorption peaks' wavelength of the CeF_3_:Mn NCs are in good agreement with those reported for CeF_3 _bulk crystals [22]. The CePO_4_:Mn NCs exhibit two absorption bands with peaks at 256 and 273 nm [23]. The two bands are overlapped because the excited state is strongly split by the crystal field [24]. We note that the Mn^2+ ^^6^A_1g_(S)-^4^E_g_(D) and ^6^A_1g_(S)-^4^T_2g_(D) absorption transitions from 310 to 350 nm [18] in these NCs are not obvious due to the much weaker Mn^2+ ^absorption ability and low Mn^2+^/Ce^3+ ^ratio in the host.

### Photoluminescence properties

**Figure 4:**
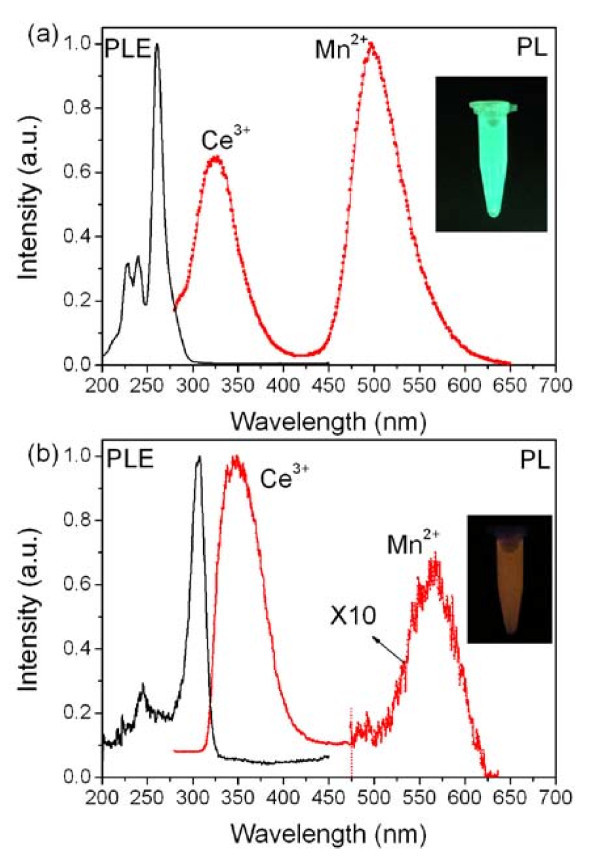
**PLE and PL spectra**. PLE and PL spectra of CeF_3_:Mn (**a**) and CePO_4_:Mn (**b**) NCs.

Figure 4a schematically depicts the Ce^3+^-Mn^2+ ^energy transfer process in the CeF_3_:Mn NCs, which efficiently induces a bright green luminescence under UV irradiation at RT. The RT PL emission spectra (with excitation wavelength *λ*_ex _= 260 nm) of the CeF_3_:10%Mn NCs contain not only the strong Mn^2+ ^emission at 498 nm but also the Ce^3+ ^emission at 325 nm. As known, the Mn^2+ ^^6^A_1g_(S)-^4^E_g_(D) and ^6^A_1g_(S)-^4^T_2g_(D) absorption transition is respectively at 325 and 340 nm [18]; both of these absorption bands are overlapped by the Ce^3+ ^emission. This overlap facilitates the energy transfer from Ce^3+ ^to Mn^2+^, resulting in the characteristic ^4^T_1g_(G)-^6^A_1g_(S) emission of Mn^2+ ^[25,26]. Such Ce^3+^-Mn^2+ ^energy transfer is induced by the electric dipole-quadrupole interaction between the Ce^3+ ^sensitizers and Mn^2+ ^acceptors [19]. Furthermore, in Figure 4a, only the RT excitation peak ascribed to the Ce^3+ ^4f-5d transition can be observed at 260 nm, while the Mn^2+ ^characteristic peaks cannot be witnessed because the Mn^2+ ^absorption transitions are forbidden by spin and parity for electric dipole radiation as *T *> 200 K [27]. Since the RT Mn^2+ ^luminescence is very difficult to be found in the transition-metal concentrated materials like MnF_2 _[27], the Ce^3+^-Mn^2+ ^energy transfer offers an efficient route for obtaining Mn^2+ ^RT luminescence in nanomaterials.

Similarly, the Ce^3+^-Mn^2+ ^energy transfer process in the CePO_4_:10%Mn NCs triggers an orange luminescence under UV irradiation (Figure 4b). The emission spectra of the CePO_4_:Mn upon excitation at 260 nm contain both the Ce^3+ ^emission at 355 nm and the Mn^2+ ^orange emission around 575 nm arising from the ^4^T_1g_(G)- ^6^A_1g_(S) transition of Mn^2+^. As known, the luminescence output color of the Mn^2+ ^ions is strongly dependent on the coordination environment of the host lattice, such as the strength of the ligand field and the coordination number. The green emission of Mn^2+ ^ions at about 500 nm is usually obtained in a weak crystal field environment where Mn^2+ ^is usually four or eightfold [27,28]. In contrast, the CePO4 NCs have a monazite structure in which the dopant ions are probably ninefold and in a stronger crystal field environment [29]. Thus, the orange emission can be observed in our synthesized CePO_4_:Mn NCs. We note that the CePO_4_:Mn NCs synthesized are rodlike particles whose shape is greatly different from the platelike CeF_3_:Mn NCs due to the different growth behavior. To eliminate the influence of the particle shape on the luminescence output color of Mn^2+ ^ions, we have further synthesized rodlike hexagonal phase NaYF_4_:Ce,Mn NCs using our established method [21] in which the Ce^3+^-Mn^2+ ^energy transfer also results in green Mn^2+ ^luminescence at 500 nm (data not shown).

### Quantum efficiency and energy transfer efficiency

The Mn^2+ ^luminescence quantum efficiency (*η*_QE_) was determined by comparing the Mn^2+ ^emission intensity of the CeF_3_:Mn aqueous solution with a solution of quinine bisulfate in 0.5 M H_2_SO_4 _with approximately the same absorption at an excitation wavelength of 260 nm [30]. It is important that all the sample solutions were sufficiently diluted (absorption value of 0.03 at 260 nm) to minimize the possible effects of reabsorption and other concentration effects [31]. The *η*_QE _of the CeF_3_:Mn NCs increases significantly and reaches 14% as the doped Mn^2+ ^molar concentration increases to 2%. The decreased *η*_QE _at Ce^3+ ^concentrations above 2% is probably due to the increased Mn^2+^↔Mn^2+ ^energy migration which weakens the Ce^3+^-Mn^2+ ^energy transfer. We note that the highest *η*_QE _we obtained is similar to that of the Ce, Tb co-doped LaF_3 _NCs reported previously [32].

**Figure 5:**
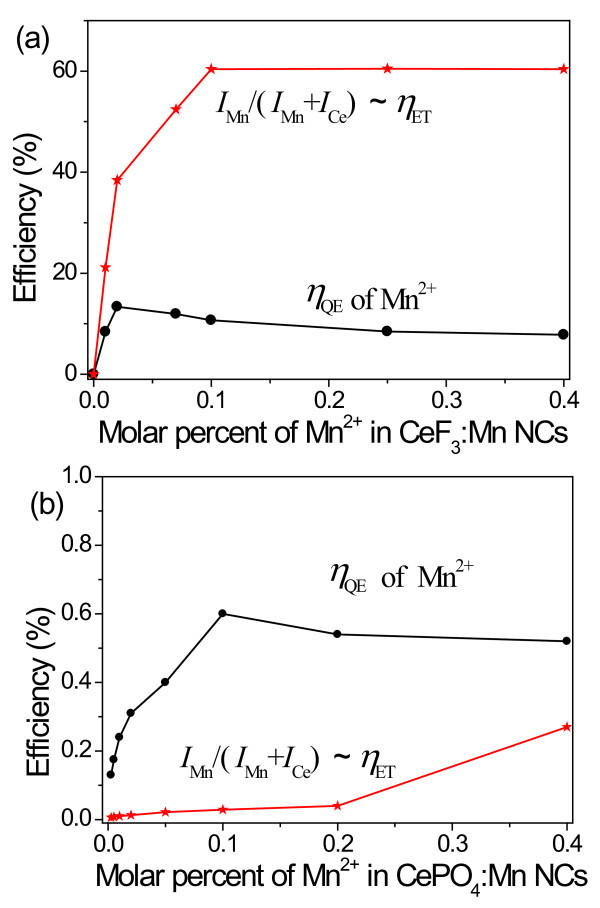
**Investigated *η***_**QE **_**and *η***_**ET**_. Mn^2+ ^luminescence quantum efficiency (*η*_QE_) and Ce^3+^-Mn^2+ ^energy transfer efficiency (*η*_ET_) vs. molar percent of Mn^2+ ^in CeF_3_:Mn (**a**) and CePO_4_:Mn NCs (**b**).

The Ce^3+^-Mn^2+ ^energy transfer efficiency (*η*_ET_) was estimated from the emission intensity ratio *I*_Mn_/(*I*_Ce _+ *I*_Mn_) when the sample solutions were sufficiently diluted and the energy loss caused by the re-absorption effects between different particles could be neglected [31,33]. As shown in Figure 5a, a high *η*_ET _of 60% is observed in the CeF_3_:Mn NCs while the Mn^2+ ^doping concentration is over 10%. We note that the *I*_Mn _is much weaker than the *I*_Ce _in the previously reported Mn,Ce co-doped CaF_2 _and other bulk materials because of a portion of randomly dispersed Ce^3+ ^and Mn^2+ ^ions beyond the interaction distance for the short-range energy transfer [19,34]. In our CeF_3_:Mn NCs, the Ce^3+^-Mn^2+ ^clusters are easily formed and result in the efficient Ce^3+^-Mn^2+ ^energy transfer.

By using the method discussed above, we have also investigated the *η*_QE _and *η*_ET _of the CePO_4_:Mn^2+ ^NCs in the presence of different Mn^2+ ^concentrations (Figure 5b). Upon doping with the increasing concentrations of Mn^2+^, both the *η*_QE _and *η*_ET _increase firstly, and the *η*_QE _reaches the peak at 0.6% when the Mn^2+ ^doping concentration is 10%. It is worth noting that both the *η*_QE _and *η*_ET _in the CeF_3_:Mn NCs are higher than those in the CePO_4_:Mn NCs. Compared with phosphates, fluorides normally have lower vibrational energies, which can decrease the quenching of the excited state of rare earth ions [35] and result in higher quantum efficiency. Besides, the energy transfer efficiency between the sensitizers and acceptors is influenced greatly by the interaction distance of these dopant ions [19,36]. Here, the less energy transfer efficiency in CePO_4_:Mn is probably attributed to the larger interaction distance between the Ce^3+ ^and Mn^2+ ^ions. A further increase of the quantum efficiency and energy transfer efficiency is possible by applying an undoped inorganic shell as a protective layer.

## Conclusions

The sensitized Mn^2+ ^luminescence has been realized based on the Ce^3+^-Mn^2+ ^energy transfer in the prepared Mn^2+^-doped rare earth NCs. The ^4^T_1g_(G)-^6^A_1g_(S) characteristic emission of Mn^2+ ^reveals green luminescence in CeF_3_:Mn and orange luminescence in CePO_4_:Mn, resulting from the crystal field differences of these two hosts. We worked out that the highest Mn^2+ ^luminescence quantum efficiency can reach 14% and 0.6% in the CeF_3_:Mn and CePO_4 _NCs, respectively. Our results may find applications in the manipulations of the Ce^3+^-Mn^2+ ^energy transfer for redox switches [37] and broadly impact areas such as photonics, light-emitting diodes, and bioimaging based on manganese materials.

## Authors' contributions

YD carried out the photoluminescence property studies and drafted the manuscript. LBL participated in the revision of the manuscript. ML and DF He participated in the synthesis of the nanocrystals. LX and PW contributed to characterization of the nanocrystals. XFY conceived of the study, and participated in its design and coordination. All authors read and approved the final manuscript.
